# A Theoretical Comprehensive Framework for the Process of Theories Formation

**DOI:** 10.1155/2021/5074913

**Published:** 2021-11-28

**Authors:** Goded Shahaf

**Affiliations:** The Applied Neurophysiology Laboratory, Rambam Health Care Campus, Haifa, Israel

## Abstract

Scientists rely more and more upon computerized data mining and artificial intelligence to analyze data sets and identify association rules, which serve as the basis of evolving theories. This tendency is likely to expand, and computerized intelligence is likely to take a leading role in scientific theorizing. While the ever-advancing technology could be of great benefit, scientists with expertise in many research fields do not necessarily understand thoroughly enough the various assumptions, which underlie different data mining methods and which pose significant limitations on the association rules that could be identified in the first place. There seems to be a need for a comprehensive framework, which should present the various possible technological aids in the context of our neurocognitive process of theorizing and identifying association rules. Such a framework can be hopefully used to understand, identify, and overcome the limitations of the currently fragmented processes of technology-based theorizing and the formation of association rules in any research field. In order to meet this end, we divide theorizing into underlying neurocognitive components, describe their current technological expansions and limitations, and offer a possible comprehensive computational framework for each such component and their combination.

## 1. Introduction: The Principles of Theorizing and Its Comprehensive Generalization

The wisdom of building and improving theories regarding our environment is a peak cognitive achievement of our human society. In this context, a theory could be described as the identification of a set of association rules, among the imprints of the world reality upon our senses [1]. Notably, complex theories about our environment are often the accumulative product of a group effort and not of a single brain. Furthermore, their development may also be based upon technological aids including, nowadays, modern computerized intelligence.

In fact, it seems that we are in an era, in which the process of theorizing, and specifically of scientific theorizing, will involve, in an ever-growing manner, computerized intelligence [2]. On the one hand, this growing involvement offers great potential for extracting effective theories from vast and complex data sets. But, on the other hand, at times, it seems that the computational methods employed might be complex and not intuitive (e.g., [3]) and may lead to less-informed reliance upon methods, which, even if powerful, could still limit the scope of possible results and enable, or prefer, the formation of only specific types of theories or sets of association rules.

Still, as a product of the human brain (or of a society of brains), it should be possible to describe theory building in terms of a neurocognitive computational process or as an algorithm [4]. Generally, neurocognitive processing has computational limitations, and the neurocognitive algorithmic description of theory building is no exception in this regard. Like any other “neurocognitive algorithm,” it should have computational limitations. Furthermore, even a society of such embodiments, which further utilizes external computational aids, would still have computational limitations.

Thus, the purpose of the current work is to present a preliminary analysis of the “algorithmic” components and flow of the neurocognitive processes, which underlie theory building, and to discuss their current and possible futuristic computerized extensions. Once such an algorithm of theorizing is formulated, including such current and potentially futuristic computerized extensions, it may contribute, on the one hand, to our theorizing ability and, on the other hand, might shed light on specific artificial intelligence methods, which are used in theorizing their potential role, their merits, and their limitations.

Therefore, as stated, the core of this manuscript is a neurocognitive analysis of the process of theorizing. To this end, we start by noting that our ability to identify association rules (or to theorize) is based, as a first step, on our sensation of the environment, which involves the formation of brain representations for the environmental stimuli that act upon us. Furthermore, the association between representations is based on the co-occurrence of their activation. However, the evaluation of co-occurrence of representation activations is based on some “indexing” of the activations, for example, in time or in space. Then, activations, which occur within a certain proximity window (e.g., in time or in space), could be considered as co-occurring and could be associated. Therefore, the first major component of theorizing, which will be presented in some detail below, comprises sensory activation of stimulus representations and their indexing.

Upon this initial major component of sensation and indexing, the brain associates between the sensory representations on the basis of the co-occurrence of their activations. In fact, the brain can associate two or more representations. Therefore, we will discuss below, in some detail, a second major component of the association between sensory representations, based on their co-occurrence in a given sample of the environment. We will term this major component of association: the “bottom-up” association, as it generates the association rules from the elementary sensory representations.

Still, it seems that a major part of our identification of the existence of association rules within a given sample of the environment is based on the evaluation of the applicability (and possible adjustment) of previously learned association rules. Often, the current sample might be too small for generating strong new associations (an underfitting error). Furthermore, we hope that associations, which are generated on the basis of a specific sample, might be relevant for other samples, at least from similar environments, in order to enable prediction and favorable intervention. However, in a given (and especially in a small) sample, there is also a risk for specific (nongeneralizable) co-occurrences, which take place either by chance or due to specific circumstances during the sample (an overfitting error). Lastly, the thorough sample-based bottom-up association is demanding computationally. Often, it is less consuming to explore top-down the applicability of previously learned association rules.

Therefore, we will also discuss below, in some detail, a third major component of evaluating the applicability of previously learned association rules for the current sample. We will term this component of association by checking the applicability of previously learned association rules: the “top-down” association, as it starts from the rules and looks for their manifestations in the currently sampled elementary sensory representations. Notably, the previously learned association rules are simply the result of earlier samples or their combination. So, in a sense, the top-down association is an exploration of association rules, which are shared by multiple samples (sample-crossing association rules). Importantly, bottom-up and top-down associations may be combined. For example, after partial bottom-up processing, the applicability of top-down association rules might become more evident. Furthermore, we will also discuss below the impact of the association components on the first component of sensation and indexing.

To summarize, it might be possible to view theorizing as being comprised three major components: (1) sensation and indexing, (2) bottom-up association, and (3) top-down association and derived reshaping of sensation. Below, we will discuss each of these major components in some detail, as well as the interaction among them. For each major component, we will discuss its underlying components, which together characterize the “algorithm” by which our brains identify the association rules or theorize. As was stated above, complex theories are often not the product of a single brain, but rather of a society of brains. Furthermore, we use various external aids, for example, paper and pencil as well as advanced artificial intelligence, in the process of identifying the association rules. Therefore, our algorithmic characterization will also encompass these aspects of theorizing. Finally, for various algorithmic components (except for the elementary sensory level, which might be viewed as a limit given, upon which our theories are formed), we will discuss the possible principles of their comprehensive computational expansion.

It should be emphasized that the current manuscript does not suggest an implementational algorithm. It seems that the abstract comprehensive algorithmic components, which are suggested below, would be, even if feasible to implement, very demanding in terms of computer resources. However, on the one hand, computer resources are constantly improving, and on the other hand, it might be possible to develop practical implementation embodiments of the algorithmic components described in this work. Furthermore, even a strictly theoretical and impractical description might contribute to our understanding of the theorizing process and the current merits and limitations of its computerized expansions. Therefore, we do not present here a detailed algorithmic description but halt at the level of basic numerical examples for the sake of illustration. The numerical examples are given in “demonstration boxes” for the sake of concretization of the more complex components. However, we have learned that for some readers, the basic text, without the demonstration boxes, might be sufficiently clear.

## 2. The First Major Component: Sensation and Indexing (The Data Set for Association)

**Claim 1 .**

The underlying substrate of our theorizing or association processes is the activations of sensory representations of the world. These representation activations could be computerized in a comprehensive manner, and in fact, computerization may enhance this set of representation activations for the sake of association and theorizing.

Suggested subcomponents for modeling sensory representation are as follows:

1. The elementary sensory layer and its dimensions
2. The array of the compound entities of association
3. The values set per entity
4. Indexing entities

### 2.1. The Elementary Sensory Layer and Its Dimensions

**Claim 2 .**

The elementary substrate of our representation of the world involves activations of our sensory layer. These activations could be viewed as involving discrete representations with the following four dimensions: (1) the discrete values of a specific physical attribute, which is being activated (e.g., specific ranges of light wavelengths, specific ranges of temperature, specific ranges of sound wave frequencies, etc.); (2) the timing of the activation; (3) the spatial position of the activation; and (4) the intensity of the activation.

We sample the world through our senses, and the first layer of the senses comprises receptor cells, which are specialized in sensing specific physical attributes: for example, visual receptors may respond to a specific range of wavelengths in the visible light; thermoreceptors in the skin may respond to a specific range of temperatures and so on (see relevant sections in [5]). Thus, each specific set of receptors is tuned to sample a specific subset of physical attributes. Physiologically, the span of our sensory entities is diverse, yet limited in both range and resolution. There are light waves we cannot see, sound frequencies we cannot hear, and so on; there are also light waves and sound frequencies within our sensory range, among which we cannot differentiate [6]. Importantly, the limitation in resolution implies that the range of values we can sense, in a given elementary sensory set of receptors, is discrete. Indeed, as each specific set of receptors, which sense specific physical attributes (light wavelength, temperature, sound wave frequency, etc.), comprises a limited number of sensory neurons, which are often active in a binary mode (all-or-none action potentials), it is reasonable to expect that the sensation of each such physical attribute would involve a limited set of discrete values, and each value would involve a limited set of discrete intensities of activation.

The activation of the neurons of each sensory set, by a transient stimulus, is limited in time and is often manifest by a burst of activity (e.g., action potentials), which lasts for a limited duration, for example, less than a second [7]. Thus, each activation of a sensory set has also a temporal (timing) value. Furthermore, the receptors are often sensitive to the spatial location of the stimulus (e.g., its place in the visual field or its somatosensory location on the body surface) [8]. Therefore, it is possible to view each representation, which is evoked at our elementary level of sensation, as comprising four dimensions that are: the physical attribute value, its intensity of activation, the time of the activation, and the spatial position of the activation.

**Claim 3 .**

The use of external technology-based sensors seems to expand our sensory layer. For example, it seems possible to sense new types of physical attributes (e.g., there are velocity and acceleration sensors instead of just time and position, which are sensed in various modalities); it seems possible to expand our sensory range (e.g., by infrared or ultraviolet sensors); and it seems possible to increase the resolution of our sensation (e.g., by microscopes or telescopes). However, all these (hopefully useful) extensions are theory-driven and are therefore based upon associations, which were built from our elementary sensory layer.

Allegedly, the limited range and resolution of our sensory abilities have been expanded technologically by various sensory devices, which involve receptors capable of sampling physical attributes beyond our sensory abilities. Seemingly, these devices then transform the expanded physical attributes to information, which is within the range and resolution of our sensation [9]. However, it is clear that at least certain sensory device samples are not a simple extension of our senses. Instead, they are derivatives of previous theories that combine our elementary sensations into theoretical constructs, which we then measure (and tend to treat as independent and “real” sensations): for example, velocity and acceleration are compounds of spatial position and time. Indeed, one of the greatest challenges of theorizing concerns the validity of forming such effective theoretical constructs [10].

Furthermore, even devices, which allegedly just expand the range and resolution of our elementary sensory abilities, result from previous theories that form associations between external sensors and our elementary sensory representations (e.g., transform the output of infrared sensors to our visible range of vision on the basis of a theory, which relates the activity in these external sensors and our visual system). Thus, both types of extension of our sensory abilities (“simple” range extension and addition of new constructs of physical attributes) seem to evolve from theories, which our human cognition generated from other analyses of previous samples of our environment.

**Claim 4 .**

Altogether, our ability to theorize is limited by our underlying elementary sensory layer. What we may hope for is expanding our ability to theorize within our sensory boundaries in a comprehensive manner. The current manuscript aims at providing a framework for such a comprehensive expansion of our theorizing ability.

In fact, a major outcome of this manuscript is hoped to be the presentation of a comprehensive framework for the derivation of such theories and thereby of enhanced sensation, as part of the process of theorizing. However, in accordance with the empiricist line of thought, even the most comprehensive theory is limited in its ability to model reality by our elementary sensory abilities [11]. The theories we can generate are not of reality but of its eventual sampling through our bodily sensory channels. Thus, according to the above, advanced sensing devices do not really enable any objective enhancement of our elementary sensory abilities. In this sense, they are not equivalent to our elementary sensing. Instead, they represent (hopefully) effective theories and provide effective compound representations, which are built upon our elementary sensing. The use of these compound representations may then enable even more effective association (or better theorizing). Thus, all theories could be viewed as based upon our set of limited elementary sensory representations on its four dimensions: physical attribute values, their intensity of activation, the time of the activation, and the spatial position of the activation.

### 2.2. The Array of the Compound Entities of Association

**Claim 5 .**

Our brains build compound representations by associating the elementary representations from the elementary sensory layer in a hierarchical manner. The formed representations are arranged within a limited and hierarchical set of modules, whereby each module includes related representations of a specific physical attribute: for example, the representations in the “lines” module may relate to the perception of alternative line tilts, while the representations in the “words perception” module may relate to alternative word perceptions.

The sensation could be viewed as comprising modules, which are arranged in tiers. Thus, despite some top-down modulation [12], the organization of the sensory nervous system, beyond its elementary level, could be viewed as hierarchical, whereby each tier comprises modules, which combine information from specific modules of underlying tiers. For example, in the visual channel, the hierarchical organization involves at the elementary tier sensory modules, which sense specific ranges of wavelengths (the visual physical attribute values); then at the following retinal tiers, there is the processing of color contrast, by differentiating information from two different types of wavelength receptor cells, brightness; by combining information from various receptor cells, the transience of the stimulus; and by differentiating information over a time unit [13]. This hierarchical combination continues all the way through the visual cortex, which comprises modules with several levels of hierarchy that combine information about line shapes, angles, color, movement, and so on [14]. Thus, the various tiers form a hierarchical set of sensory modules, which process physical attributes of varying complexity. Indeed, this organization of a hierarchy of specific sensory modules also applies to the other (nonvisual) sensory channels (e.g., [15]). Furthermore, while more basic modules relate to a specific sensory channel (unimodal), the highest modules combine representations from multiple sensory channels (heteromodal) [16].

**Figure 1:**
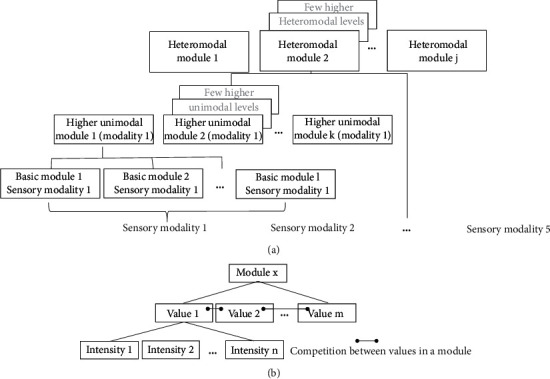
The structure of representation of sensations in the brain. (a) Stimuli are perceived via the various sensory modalities. In each modality, a stimulus activates elementary modules of representation. These elementary representations could be combined hierarchically to activate more complex representations, which could be unimodal (belong to one sensory modality) or heteromodal (combining representations in various sensory modalities). The depth of the hierarchy, both unimodal and heteromodal, is rather limited. Also, the number of different modules in the brain is limited. (b) Any module (elementary, higher unimodal, or higher heteromodal) comprises values, which compete among themselves by a mechanism of lateral inhibition. Each of these values (e.g., different faces in the “faces module” or different line tilts in a more basic “lines module”) is activated at any given time with a certain level of intensity out of a discrete set of intensity levels.

However, altogether, the number and complexity of modules, in our sensory hierarchy, are rather limited [16].  Figure 1(a) presents this structure schematically. Importantly, such a well-structured hierarchy promotes certain types of preferred associations: for example, faster perception of words from combining specific auditory patterns. However, on the other hand, it poses limitations and reduces the priority of other possible, less hierarchical, associations: for example, between representations in different sensory modalities. Certainly, we can identify also associations between value representations in modules, which are not related by hierarchy. However, such hierarchy crossing association requires the recruitment of cognitive processes, such as working memory, which are of limited capacity.

**Claim 6 .**

The compound representations within each of the physical-attribute modules are based on activations of neuronal networks. Similar to the representations at the elementary sensory level, these activations could be viewed as involving the following four dimensions: (1) the discrete values of a specific physical attribute, which is being activated (only this time more complex attributes than at the elementary sensory level); (2) the timing of the activation; (3) the spatial position of the activation; and (4) the intensity of the activation.

Each module in the hierarchy is a collection of neuronal networks, which may represent alternative, but related, sensory objects. For example, alternative faces are represented in the “faces module,” and alternative line tilts are represented in a more basic “lines module.” These neighboring networks, with alternative value representations, compete among themselves through a principle termed lateral inhibition. According to this principle, each active representation inhibits (reduces the activation of) other representations of alternative sensations within the same module, thereby rendering a sharper selection of the specific stimulus representation (the “winners-take all” principle) [17]. Thus, the representation of sensory stimuli is organized in modules, which comprise alternative values (embodied by different neuronal networks in the same module).

During its activation, the neuronal network, which represents a specific stimulus, generates a “burst” response. In this burst response, multiple neurons fire in synchrony. This burst could be graded in terms of the intensity of network activity and with some variability in duration (often under the 1 s time scale) [18]. Thus, if a stimulus is sensed more intensely, a stronger response could be evoked by the relevant network. However, the network output activity (the activity, which is sensed by other network units and can be used for association) is a summation of the activity in a limited number of the network's output neurons. Furthermore, the single neuron activation is binary (on-off and all-or-none). Therefore, taking together the limited number of output neurons per network and the binary activation of each such neuron [19], there is a limited set of discrete possible intensities of output activation of the neuronal network. Thus, it is possible to consider the representation of each such neuronal network as comprising a range of discrete (even if at times possibly multiple) intensities (Figure 1(b)).

Thus, similar to the elementary representation level, also at higher levels, the brain represents stimulus features (values) and their intensity. Furthermore, as was stated above, also at the higher levels of representation, the activation of each representation, by a transient stimulus, is limited in time and is often manifest by a burst of activity (action potentials), which lasts less than a second. Thus, each activation of a sensory set has also a temporal dimension (its timing). Furthermore, the hierarchical summation, by which values at a given level are based upon values of lower levels, tends to be spatially oriented, namely, to associate values from proximal locations [20]. Therefore, at least many of the value representations in the sensory hierarchy also have a spatial dimension (position). Thus, altogether, similarly to the representation at the elementary level, it is possible to view each representation in the sensory hierarchy as comprising the same four dimensions, which are: a specific stimulus value (specific face, specific line, specific word, etc.), its intensity of activation, the time of the activation, and the spatial position of the activation.

**Claim 7 .**

Current technology can already support the comprehensive removal of hierarchical constraints upon representation associations.

**Figure 2:**
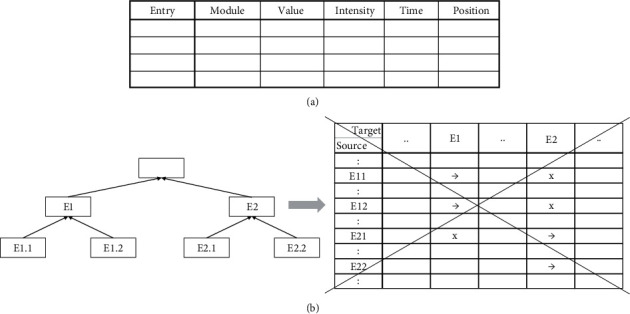
Replacement of the brain's hierarchical representation of sensation by a general table description. (a) The top table has one entry for each activation of representation anywhere in the sensory hierarchy, which has, as described in the text, the dimensions of value, intensity, time, and position. (b) The hierarchical relation between these table entries could be described by a matrix of relations, which may enable some relations (√) and disable others (X; degree of enablement was ignored for simplicity). However, it is possible to ignore the matrix limitations and permit association between any pair or set of entries.

It seems that the hierarchical organization of representations in the brain could pose significant limitations to the association process. However, we can in principle describe the hierarchical representation of the brain in a table format (Figures 2(a) and 2(b)) and, in fact, can cancel the hierarchy altogether (Figure 2(b)).

In the table format, the discrete combinations of values and intensities, which were described in the brain representation, could be replaced by combined new values. Thus, there is no a priori need to select only one intensity activation for a given brain value. Furthermore, the entire hierarchical structure could be cancelled, and thus, all associations between entities and values would be feasible, without hierarchical limitation. For the sake of further abstraction away from the strict brain structuring, we would also replace the term brain module with the more abstract term entity. Thus, we can talk about the values of entities instead of the intensities of values of brain modules.

However, one likely advantage of the hierarchical brain representation is that a higher-order value could be activated, even when only some of its underlying basic values were activated. Provided that this partial activation of basic values crosses a combined threshold for activating the representation of the higher value. For example, we need not see, in full detail, both eyes, nose, mouth, and so on in order for a specific face representation to be activated. This enables flexibility in the sensation of “real-life” environments, in which there are multiple alternative combinations for basic value sets, which may activate a higher module value [21].

In a tabular nonhierarchical organization, there would still be use for compound entities (note: an “entity” is the general term we use in the switch from a concrete brain module to an abstract description), which comprise various partial combinations of more basic entities, for the very same reason of enabling tolerance in representation. Similar to the higher hierarchical modules in the brain, these compound entities are the result of association (also emerging from previous samples, as will be discussed below, by ways of bottom-up and top-down association). However, the compound entities would not necessarily form a hierarchical structure, but rather a more flexible, heterarchical structure. Indeed, such a heterarchical structure might capacitate many more entities (which are formed by association) in comparison with a strictly hierarchical structure. An ever-increasing computational power may enable this increased capacity.

### 2.3. The Values Set per Entity

**Claim 8 .**

On top of the limited number of entities (brain modules), the brain also has a limited capacity of values per entity.

We may feel we are capable of representing entities with an immense number of values, like the words in a language, or even an infinite number of values, such as the natural numbers. Yet our representation of entities with infinite values (e.g., the natural numbers) or entities with an immense number of values (e.g., the words in a language) comprises sequences of basic entities (see, e.g., relevant sections in [22]). For example, natural numbers comprise sequences of digits, each with ten possible values, and words in a language comprise sequences of letters. The number of values in a brain set (entity) is limited, and the associations we generate between these values, in different entities or sets, are based on a sequence of associations between the basic values, which underlie them. For example, the arithmetical operations, which associate between two numbers and their sum, multiplication, or any other mathematical output, are merely a sequence of associations between the digits of these two numbers and the digits of the output [22].

**Claim 9 .**

As stated above, technology can expand the number of possible entities. In principle, technology can also expand the number of possible values per entity. However, a large number of entity values may mean a small number of occurrences per value. As the association is based on co-occurrence, reduced occurrences per value may hinder it. Therefore, it might be advisable instead to continue the use of a limited number of basic entity values.

When we discussed above the array of compound entities, we emphasized the merits of the increased capacity, which is made possible by the technological expansion that promotes the heterarchical and comprehensive formation of compound entities from the elementary sensory representations. Allegedly, it might have been useful to expand similarly the capacity of value representations per entity, and indeed, this seems to be done by some computational tools. However, in effect, such computational tools involve specific operations (e.g., mathematical operations, lexical operations, etc.), which, as we stated above, are based on sequences of operations on the basic entities (digits, letters, etc.) that underlie the multivalue entities.

Indeed, when we look for associations, which depend on a sufficient count of co-occurrences, as is the case with our brain-based intelligence, we need sufficient counts of occurrence of the to-be-associated values. The capacity increase, which is enabled by computation, could also increase the number of possible entity values. However, if there are too many values for given sample size, the count of single value occurrences (and, therefore, the count of co-occurrences in which these values participate) might be too small for forming associations.

Therefore, we do not necessarily seek to increase the number of possible entity values. Instead, it seems that a comprehensive expansion of the specification of a number of values per entity would better be based upon the division of this entity into basic entities, such as digits for numerical values and letters for words. The precise determination of the number of basic entities (and the number of their possible values), which should be used to span a given entity, could be dynamic. It could be determined on the basis of associations found in the current and previous samples. The associations formed between the basic entities could then be combined to form more complex associations between the given entities, as will be described below, for the bottom-up and top-down association components.

### 2.4. Indexing Entities

**Claim 10 .**

Association between entity values in the brain is based upon temporal proximity and often also upon spatial proximity. Therefore, it is possible to consider the activation of each entity value in the brain as indexed in time and often also in space.

As was suggested above, the burst activation of each value in any module often lasts well below one second. However, the association is based on co-activation [23]. Thus, it is possible to derive a temporal “index” of activation, which defines how proximal in time should the activation of two or more values occur in order for them to associate. Certainly, the brain has the ability to reverberate the activation of various sensory representations, for example, over multiple seconds with the mechanism of working memory. However, the capacity for co-activation of multiple representations, by such mechanisms, is rather limited [24].

Furthermore, the brain representation is also tuned for spatial indexing. Thus, various modules of representation, from the elementary (receptor) level and upward, are sensitive to space (e.g., position in the visual field or somatosensory positioning over the body). With the advancement in the sensory hierarchy, there seems to be a gradual blurring of spatial precision [16]. Yet, even at relatively higher levels, representations of spatial proximity tend to associate more than representations from distant loci. Thus, it is possible to state that brain representations are indexed for association in time and in space, with some degree of temporal and spatial tolerance.

The brain also possesses some ability to use various modules, other than time and space, as an index for association [25]. For example, we can explore for associations between characteristics of people (or of any other group of objects). The grouping characteristics (people) are, in this case, the indices, and the different characteristics are the representations, which are being associated, such as height and weight. Both the grouping characteristics and the associated characteristics are represented in the brain as values of specific modules, or their combinations, to begin with. Usually, these indexing modules would be of higher levels in the sensory hierarchy. Still, the value modules in the sensory hierarchy, which are beyond the elementary level, are built by the association between elementary-level values on the basis of their temporal and spatial proximity (or, in other words, based on their similar temporal and/or spatial indexing). For example, people are perceived as people in the first place because of spatiotemporal proximity in the activation of representation of their body parts, and therefore, the association of their height and weight is also based on spatiotemporal indexing, to begin with. Generally, the mere representation of values, beyond the elementary level, is already based on temporal and spatial indices. Therefore, even such allegedly nontemporal and nonspatial indexing, by the brain, is based upon indexing modules, which are based upon temporal and/or spatial indexing. Thus, temporal and/or spatial indexing underlies it and any kind of brain-based association. It seems challenging to even imagine modeling our world without a strong preference for associations between sensory activations, which are proximal in time and/or in space.

**Claim 11 .**

Technology may enable overriding the time- and space-based indexing limitation for the association, using also the other representation dimensions of a physical attribute value, intensity, or any combination of the four representation dimensions (time, space, physical attribute value, and intensity) for indexing

As was stated above, our brain is capable of utilizing nonspatial and nontemporal indices. However, these indices would be represented in higher levels of the sensory hierarchy, and therefore, their very formation is based upon temporal and spatial indexing.

Technology enables further indexing flexibility with any entity or any combination of entities in the general tabular structure of the sensory data set, which was described above (see Figure 2 and related text) [26]. Demonstration box 1 presents the potential value of indexing by a specific entity for the identification of association in a finite sample. However, it should be remembered that the entities and values in the tabular data set are still the results of our brains and, thereby, as described above, were formed on the basis of temporal and spatial indexing. Thus, even the technological expansion of our indexing ability still embeds temporal and spatial indexing.

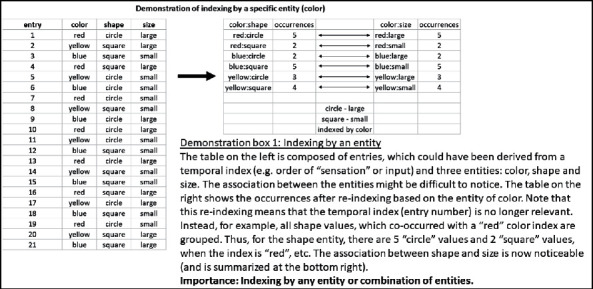

But, furthermore, the example in demonstration box 1 of association by color may also be viewed as a demonstration of a potential qualitative expansion beyond the basic brain's ability. As was discussed above, our brains associate on the basis of temporal and spatial indexing. We further noted that higher sensory representation modules (or entities), in the brain, are also formed on the basis of this temporal and spatial indexing, and therefore, their use as indexing entities already embeds temporal and spatial indexing. However, we also realized that at the level of the elementary sensory representation, time and space are merely two of the four sensory dimensions, which also include the physical attribute values and this value's intensity. At this elementary level, there is no a priori preference for the representation dimensions of time or space over the representation dimensions of physical attribute values or the value intensities. Each of these dimensions, or their various combinations, could be used equally for indexing. Therefore, demonstration box 1 could also be viewed as an example, in which the physical attribute of color (sensed by wavelength receptors at the elementary sensory level) was used for indexing, instead of the activation timing (or order). Altogether, we may also embody a significant external expansion of our association ability by indexing on the basis of the other dimensions of physical attribute values and value intensities and need not be limited by the brain's tendency to index by time and space.

## 3. The Second Major Component: Bottom-Up Association (Intrasample Association)

**Claim 12 .**

The first type of the association we discuss is “bottom-up” from the sampled data to association rules. It is limited, both in our brain and with currently available technologies, in terms of capacity (e.g., number of possible entities and values, which could be associated) and demand for temporal and spatial proximity to evoke association. These limitations could be overcome in a comprehensive manner.

Suggested subcomponents for modeling bottom-up association are as follows:

1. Indexing association
2. Multientity bottom-up association

### 3.1. Indexing Association

**Claim 13 .**

The formation of association between representations is restricted by the proximity of their temporal and spatial indexing. While the brain and technological expansions permit for some tolerance in the required index proximity, it is still limited.

As was presented above, our sensation of the environment is indexed in time and space. Furthermore, we suggested that the brain possesses innate, yet limited, abilities for temporal and spatial tolerance, which could be used in the process of association between values. As we discussed, even when higher-level entities are used by the brain for indexing, instead of proximal time or space relations, these other entities were probably formed on the basis of proximal time- and space-based associations (from the values of entities at a lower level in the sensory hierarchy). Thus, altogether brain-based association is heavily based on proximal time and space indexing.

Using computational expansion, the brain tolerance in indexing could be increased. For example, with methods of spatiotemporal data mining, it is possible to associate between distant (in space and in time) entity values and also to associate between values with large tolerance regarding the precision of their (spatiotemporal) distance [27]. Still, the expansion offered by current computational methods involves limiting assumptions regarding the spatiotemporal distance between the associated values and regarding the tolerance of the association window.

**Claim 14 .**

Technology may enable overriding the indexing proximity constraints in a comprehensive manner.

However, theoretically, the association needs not be limited by ranges of index proximity between the associated entity values or by the degree of tolerance regarding their relative distance. It could be possible to associate between activations, which are indexed differently and apart from each other, without any a priori limitations, as long as the indexing of the two entities relates by some repetitive law. For example, values of one entity at given index entries (denoted by *i*) could be associated with values of another entity at the succeeding index entries (*i* + 1) or with values of yet another entity at the double entries (*i* × 2). In order to perceive the feasibility of comprehensive association, between any distant index entries, it is possible to envision the index entries as a special entity and decompose it to basic index entities (e.g., the units digit entity, the tens digit entity, the hundreds digit entity, etc.). Then, it would be possible to search for associations between couples of basic entities and basic index entities on the basis of similarity in occurrence counts. As there are, for example, ten index unit values, it means that the number of samples of a couple of an index unit value and a basic entity value would be decreased tenfold on average when compared with the number of samples of just the basic entity value. However, in a sufficiently large sample, there may still be enough counts of the couple values to form a basis for association. Demonstration box 2, part A, presents an illustrative numerical example for this possibility.

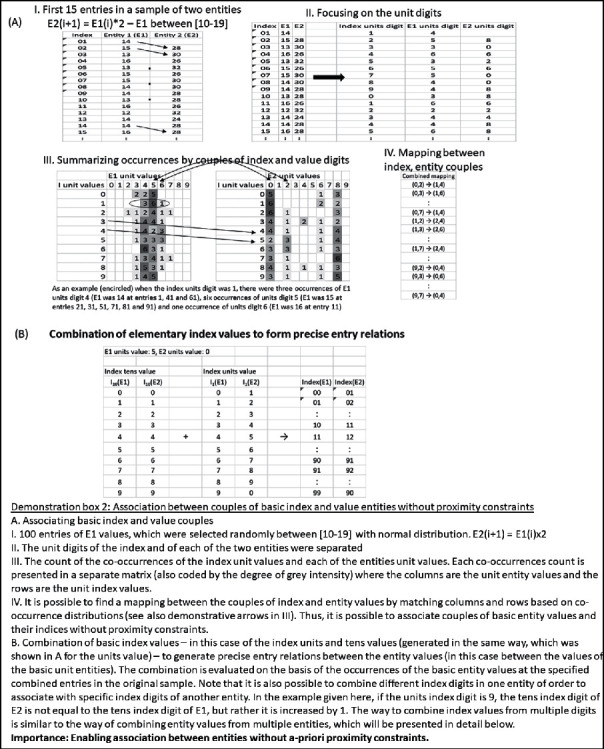

Still, after the association between the decomposed couples, there would be a need to recompose the identification of the index entries, by combining the associations of different index values and the same entity values (e.g. the index units value, the index tens value, the index hundreds value, etc.). This way a mapping could be theoretically completed, which associates values of one entity and that of another entity, without proximity constraints. Demonstration box 2, part B, continues the illustrative numerical example for this possibility.

Thus, altogether, it seems that we can envision a way to expand our ability to associate while cancelling constraints of index proximity. Each entity value could associate with any other entity value, even if their index entries are distant, as long as there is also an association between these index entry values. While there are certainly implementation challenges, the fact that we can envision such comprehensive association between index values at any distance means that it is within the reach of our extended theorizing ability.

While the origin for our discussion of unlimiting the association proximity constraints was the brain's indexing by time and space, the above principles of association without limitation of entry proximity are applicable to indexing by any entity or combination of entities, which may underlie the index.

### 3.2. Multientity Bottom-Up Association

**Claim 15 .**

Association is not just between pairs of representations but also among larger sets of representation. However, the brain is limited in its capacity for forming such set associations. Current technology enables significant expansion of this set's association capacity. But every available technology is still limited with regard to its possible and preferred associations.

As discussed above, if two representations are co-active in the brain, the connectivity between them strengthens, and thereby, they become more associated and the opposite occurs when the two representations are active separately from each other. Importantly, the associability of pairs of representations depends upon the physical connectivity between the brain modules to which they belong. Thus, pairs of representations, which belong to modules that are strongly connected, will associate more, while pairs of representations, which belong to modules, that are not so connected, will associate less effectively, given the same degree of co-activation. Nevertheless, memory mechanisms permit limited association of representations, which belong to less connected modules, by promoting indirect connectivity.

Furthermore, the association can form between groups of representations (more than just two) [28, 29]. In fact, the hierarchical structure of sensory representation embeds the association of multiple representations. For example, the representation of the face is basically an association of multiple co-occurring face parts. The association of groups of representations, which do not belong to strongly connected modules (by way of the hierarchical structure) and may rely more on memory processes, maybe even more limited than the association of only pairs of representations.

Moreover, we also emphasized above the limitations imposed upon the bottom-up association process in terms of index (temporal and spatial) distance and tolerance. Altogether, these limitations also seem graver when considering the association among groups of (more than just two) representations.

Various data mining methods are in computational use to enhance association abilities in large samples, which involve multiple entities and values. However, each data mining method has its underlying assumptions, and thereby, each such method limits the range and the preference of possible associations, which could be discovered, and even with the combination of multiple methods, there are associations that would not be reached and multiple others that would have low preferences [30]. Therefore, the challenge would be to envision a comprehensive approach, which could expose all the possible associations in a sample, without such limitations.

**Claim 16 .**

Technology may enable the scanning of all possible associations among sets of representations, without limitation.

The key for such a theoretical generalization of multiple-entity associations lies again in the finiteness of the set of discrete entity values, which was described above, or in the limited set of discrete values of the basic entities, which comprise them. For example, let us assume that a standard data mining method reached a certain formula, which associates entity *Y* with entities *X*1 and *X*2. In this case, there would be an association between the basic *X*1 and *X*2 values and the basic *Y* values (e.g., between the unit digits, tens digits, etc.). Demonstration box 3 presents an illustrative numerical example for this. However, given a sufficiently large sample, such an approach, of associating the basic values, would generate specific results for any formula of association, which would be arrived at, by any data mining method. Therefore, the approach of associating basic entity values is a conceptual way to generalize the outputs of any data mining method.

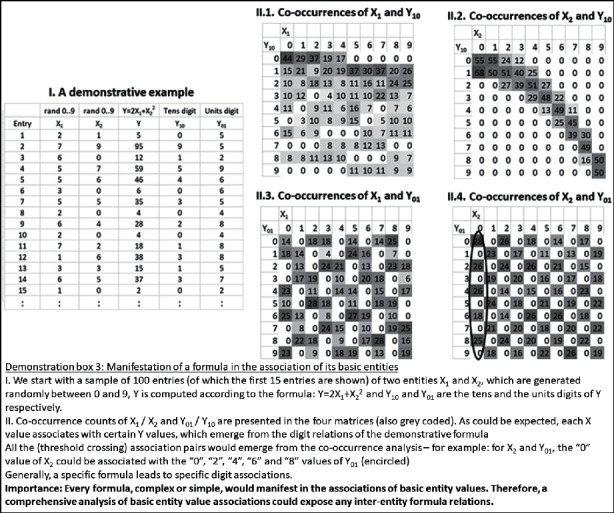

Furthermore, at least theoretically, it is possible to associate between the basic discrete values by logical AND, in which case, the *Y* value is *y* only if both the *X*1 value is *x*1 and the *X*2 value is *x*2. Demonstration box 4 presents an illustrative numerical example for this. It is also possible to associate such values by logical OR, in which case, the *Y* value is *y* if either the *X*1 value is *x*1 or the *X*2 value is *x*2. Logical OR association is also possible between two *X*1 values: for example, the *Y* value is *y* if *X*1 value is either *x*11 or *x*12. The associations may involve any number of multiple entities and entity values. However, at the level of the values, they will still follow a Boolean logic form and would be describable with AND/OR predicates. The basic items in these logical expressions would be the values or their negations (*x*1′ associates with *y* if at the given index entries, which relate *X*1 and *Y* values; the lack of occurrence of the *x*1 value, in the *X*1 entity, associates with the occurrence of the *y* value in the *Y* entity).

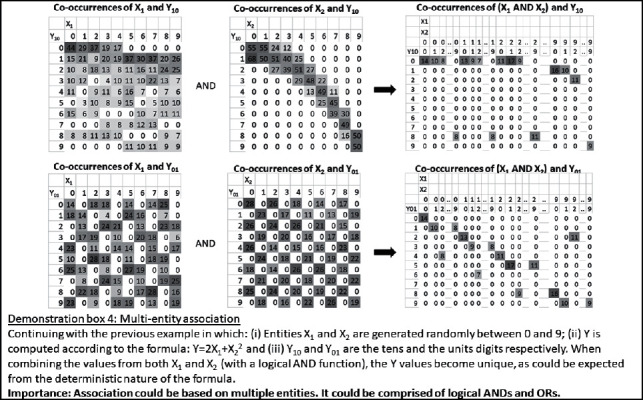

However, according to De Morgan's laws, it is possible to describe any logical expression in a disjunctive normal form, which means that the elements are values or their negations and they are combined at the first level by logical ANDs and at the second level by logical ORs [31]. Therefore, an algorithm, which aims to find all the combinations of source entity values *X*1,…, *Xn* that predict a target entity value *Y*, could search first for pair associations of *Xi* and *Y* and *Xi*' (negation) and *Y*, then search for logical ANDs between these pairs, and then form logical ORs among these AND sets. Such an algorithm will explore, in principle, all possible combinations of entity associations and thereby all multientity formulae.

Certainly, the use of a specific data mining method may enhance performance and enable the extraction of the formula from a smaller sample. However, our focus here is not on the specific implementation, but instead on the description of what a comprehensive expansion of our theorizing abilities can reach. Thus, according to the above, we can certainly envision a way to generate all possible association formulae between the values of multiple entities.

The subcomponents, presented thus far, seem to suggest a comprehensive theoretical extension to our bottom-up neurocognitive ability of theorizing. However, they might not be sufficiently effective for analyzing associations in a finite sample as they are based on discretization of the data, which reduces occurrence counts and leads to underfitting in the discovered associations. To begin with, small sample sizes certainly limit the brain, as well as the various data mining methods, in finding comprehensive associations. Furthermore, the increased discretization, suggested above for the sake of theoretical comprehensiveness of analysis, is certainly likely to reduce the efficacy of associating even further. In a way, the various data mining methods could be viewed as a means to overcome such underfitting, at the expense of using specific limiting assumptions regarding the nature of the preferred association rules. Similarly, overfitting would also be a risk in the case of small samples [32], for example, due to incidental sensory activation, which does not repeat in other samples. Indeed, this manuscript merely discusses the theoretical span of our cognitive abilities and is not focused upon the feasibility of implementation. However, we would still be interested in viewing (and in expanding) the manner by which we currently overcome these limitations.

Therefore, we should discuss and generalize also the component of correcting for this under- and overfitting. This is the goal of the next section.

## 4. The Third Major Component: Top-Down Association (Intersample Association)

**Claim 17 .**

The second type of association is “top-down,” which evaluates the applicability of predefined association rules to the sampled data. However, predefined association rules emerged from previous samples. Therefore, top-down analysis is basically the employment of sample-crossing association rules. Our brain and currently available technologies are limited in terms of the ability to scan the applicability of multiple sample-crossing association rules and their combinations. We are also limited in scanning for latent factors, which may be missing from the sample and could have improved association if sampled. These limitations could be overcome in a comprehensive manner. However, some latent factors may still stay beyond our reach.

Suggested subcomponents for modeling top-down association are as follows:

1. Top-down association
2. Latent factors search

### 4.1. Top-Down Association

**Claim 18 .**

Building associations from one sample will often be highly limited due to sample size. Instead, we are capable of looking for the applicability of predefined rules in the sampled data. These predefined rules are derived from previous samples. While our brains have only limited capacity for exploring the applicability of these sample-crossing rules, we expand our ability by harnessing ever-improving technologies for this purpose. However, even the most advanced technologies today are still based on assumptions, which limit comprehensive exploration of alternative sample-crossing rules.

In practice, we often look for associations in samples of limited size. Therefore, the number of occurrences of various entity values may be too small for the association, which is based solely upon co-occurrence. Furthermore, with small samples, there is a greater risk of underfitting as well as of overfitting. Thus, we often explore association rules using an inductive reasoning process. In essence, this process is heavily based on the exploration of the adequacy of various predefined association rules for the current sample [33]. Notably, these predefined association rules were in fact learned and established on the basis of previous samples. This exploration among prelearned association rules is guided by our prefrontal cortex with top-down exploration among the representations of entities in the relevant sensory modules [34].

Importantly, inductive reasoning is content-dependent: for example, we may explore first among a certain set of mathematical association rules if the sample is numerical and among another set of linguistic association rules if the sample comprises words. However, we are also capable of analogical reasoning [35], namely, of exploration, in a manner which crosses content boundaries and is based upon similarity of the distribution of entity values and upon similarity in the distribution of relations between entity values in the different samples [36]. Thus, analogical reasoning does not assume a priori semantic knowledge about the sample.

In fact, in a sense, inductive reasoning could be viewed as a special case of analogical reasoning. This is because each inductive rule could be described in terms of the distribution of entity values and the distribution of the interentity value relations. Thus, we can expect that if a given inductive rule is applicable in a specific sample, we would find similarity in the distribution of entity values and entity relations in the specific sample and the distribution of the entity values and relations of the inductive rule itself. For example, if the entities are numbers, we would expect to find basic entities with ten values each (digits) and relations between the values, which may relate to arithmetical operations: for example, digit multiplication by 2 would map from ten values to only five values of unit digits (even numbers only). Thus, in principle, we can describe any matching with association rules, which our brain can generate, in terms of analogical reasoning or, in other words, as being based on similarity in the distribution of entity values and value relations between samples or between samples and rules.

We further possess the ability to explore association rules by chaining several predefined rules in order to identify associations in the current sample [37]. Nevertheless, this ability of the brain to chain functions is limited in capacity [38]. Indeed, this top-down association ability of the brain, without and with chaining, is based on processes of executive function, working memory, and sustained attention, which are limited in capacity. Therefore, we often utilize external aids for the effective implementation of exploring association rules, from pencil and paper (and their historical predecessors) to advanced computing.

An advanced method for improving the human top-down exploration process for association rules is symbolic regression [39]. In its basic form, this method starts with a set of available association rules or, for quantitative data, with a set of formulae. These formulae are applied at any order on the sampled data, and effective formulae, or association rules, are maintained. Combinations of successful rules are further generated, and this hopefully leads to the selection of effective formulae, or chains of association rules, which identify associations in the sampled data. However, this process of symbolic regression is still limited, for example, by defining the set of formulae, which may be used as a priori rules. Therefore, we discuss below its possible comprehensive expansion and automation.

**Claim 19 .**

Technology may enable the exploration of all possible sample-crossing association rules.

For a given sample, every association is describable as a mapping between source and target. The nodes in the source partition are single entity values or, alternatively, the values of a set of entities, which are combined by a logical predicate, and the nodes in the target partition are other values of another entity. The edges of each such mapping represent possible association relations between pairs of source and target values. Importantly, from each sample, it might be possible to derive multiple association relations between various combinations of source and target entities. Thus, the associations in a given sample could be described by a set of such multiple and alternative mappings, among which there are partial overlaps in terms of entities, values, and association relations. The theoretical generalization of analogical reasoning would be a comprehensive exploration of similarities between such sets of mappings, which emerged from different samples. Since, as was stated above, the rules, which are evaluated for adequacy in a given sample, stem from associations in other samples. The similarity between sets of mappings could be based, in principle, only on the distribution of values and the value relations, without reference to content semantics (or to the labeling of entities and values) [40]: demonstration box 5 presents an illustrative numerical example for this possibility. As we discussed above, even content-based inductive reasoning could be viewed as a private case of content-free association. Notably, the content-free association would enable the breaking of possible barriers, which may prevent identifying certain relations between samples of only allegedly different content.

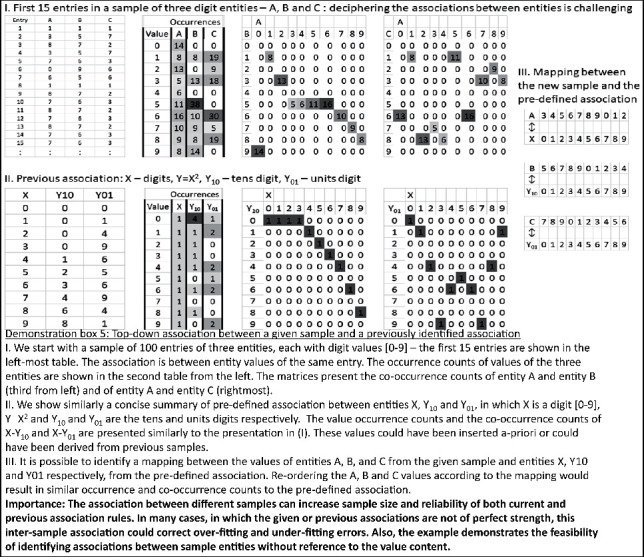

Importantly, it should be pointed out that at times, the relation between the sets of mappings might not be of equality, namely, a similar number of values in the relevant entities, or entity combinations, with similar value distributions and similar intervalue relation distributions. Instead, one set might be a subset of the other, or there might be a significant, yet still partial, overlap between the samples. These types of partial relations might also indicate under- or overfitting in the bottom-up associations of the involved samples. Thereby, such under- and overfitting could be corrected. Demonstration box 6 presents an illustrative numerical example for this possibility.

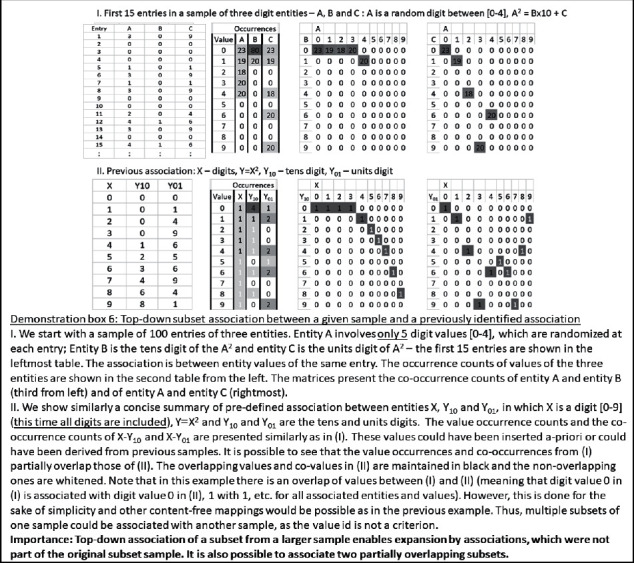

Finally, in resemblance to the brain's ability to chain rules, similarity could also associate between a certain sample and a chain of other samples. Thus, the comprehensive exploration of similarities between samples also includes sample chains. Demonstration box 7 presents an illustrative numerical example for this possibility.

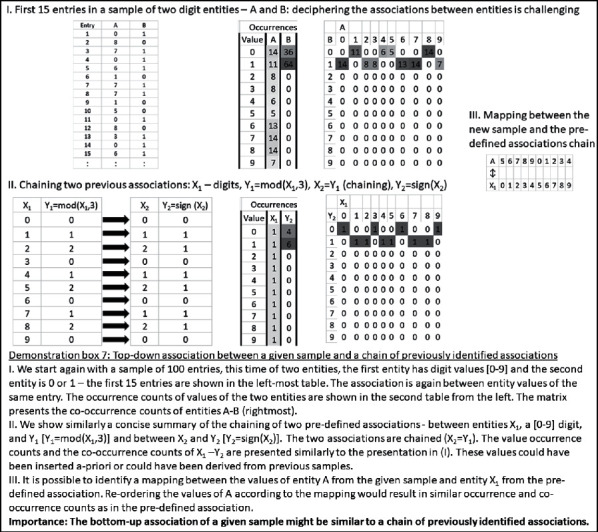

### 4.2. Latent Factors Search

**Claim 20 .**

The effectiveness of association may be limited due to the effect of latent factors (entities or values). Our brains have a certain ability to explore such hidden factors. However, it is based upon the same bottom-up and top-down components described in the previous paragraphs and is thus limited. Current technologies expand our ability in this regard but are based on limiting a priori assumptions regarding the nature of possible latent factors.

On multiple occasions, the associations for a given sample would be only partly due to the presumed impact of latent entities and values, which were not included in the analysis. It seems that the identification of such an impact and the search for latent entities and values is important for effective theory building. Indeed, studies demonstrated our ability to identify the existence of such latent factors from early childhood [41], and in the history of science, on multiple occasions, the existence of entities and values was derived theoretically first and only later was also supported by experimental data.

If the latent entities and values are present in a given sample and were simply missed in the analysis due to underfitting, we can include them for improving association precision. This condition is identified by top-down association. However, as was described above, the embodiment of top-down association by the brain is limited in its capacity.

Yet, on other occasions, the latent entities and values were not part of the sample, to begin with. Still, research regarding our exploration for such latent factors suggests we use analogical reasoning to identify similar sample sets [42]. Such exploration may identify similar sets, which may also include entities or values that improve the partial associations of the original sample. In which case, we can also try to include, with further samples from the current source, these additional entities and values. As described above, the ability of our brain for analogical reasoning is limited. Sometimes, for example, in the context of scientific theories, this analogical reasoning quest for latent factors may be described as involving intuition and insight. However, cognitive analysis of such processes seems to suggest that insight may be the emergence of the results of unconscious exploration into consciousness [43]. All in all, we have a limited ability to identify the existence of latent factors and explore them in the current sample or in other samples.

Various methods have been used to analyze latent factors [44]. Each of these methods is based on specific assumptions regarding the latent entities and values and their relation to the sampled entities and values. Furthermore, these methods are based only on the sample without automatization of the analogical reasoning of exploration for finding candidate latent variables in other samples. Therefore, below, we discuss principles for comprehensive exploration for latent entities and values, without human intervention.

**Claim 21 .**

Technology may enable the exploration of all candidate latent factors. Due to the limited elementary sensory level, some latent factors may be beyond our reach.

On the basis of our analysis thus far, our possible exploration for latent factors may be performed on three different levels: (1) among additional entity values from the current sample, which were not found in the basic association; (2) among other existing samples, which show partial similarity to the current sample; and (3) among the entities of any other possible sample out of the finite set of samples, as we will discuss below.

When the latent entities and values exist within the current sample, they may have been missed because of the computational limitations of the algorithmic implementation. It would then be possible to reexplore, among all possible basic and compound entities and values, candidates with a distribution of values that can combine with the partial association for its improvement. Thus, latent factors, which were missed could be identified in a focused analysis, which evaluates their contribution to other associations. Demonstration box 8 presents an illustrative numerical example for this possibility.

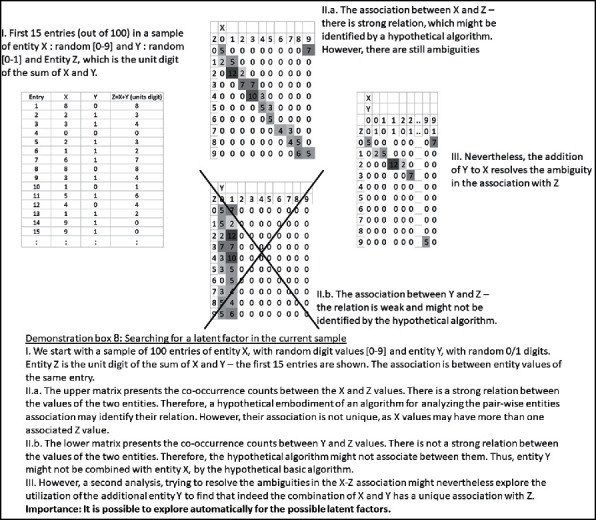

As was suggested above, the latent factor could also be explored in other samples, even if its counterpart is not found in the current sample. Thus, a partial association in the current sample might have a parallel association in another sample. However, in this other sample, there might be an additional entity, which could be added to improve the association. In which case, it might be possible to explore the origin of the current sample for entity values, which could parallel to the additional entity. Demonstration box 9 presents an illustrative numerical example for this possibility.

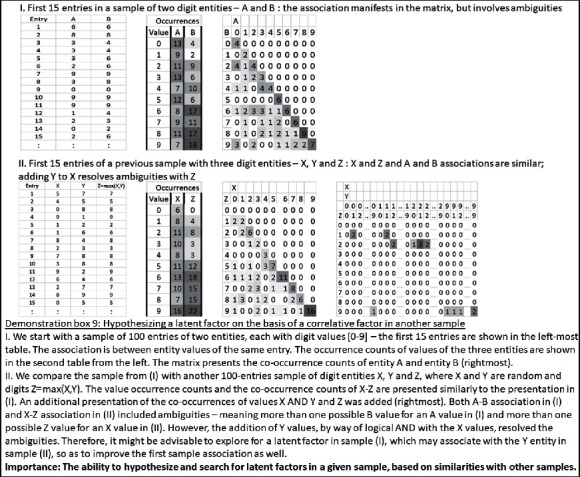

Furthermore, as described for the top-down association, similarity could also be analyzed, for the purpose of identifying candidate latent factors, between the partial association sample, on the one hand, and a chain of other samples, on the other hand.

Note that given a large enough set of samples and their derived association rules, currently prevailing methods of exploring latent factors (e.g., hidden Markov models as one example) would emerge, as a special case. This is because they are merely based on a sequence (chain) of arithmetic operations (associations), and as we described above, each such association is based on a small set of relations between basic entities (or, in the arithmetic case, of digits). However, if the previous sample set is large enough, such underlying associations are likely to be already included in it. In fact, the view of the advanced methods, as composed of chains of associations, relates to a core aspect of this work. Arithmetic formulae, like any other association rule in any domain, comprise a limited set of underlying associations (addition, subtraction, multiplication, division, etc.) and their chaining.

Finally, it is possible that a latent factor is not found in the current sample, or in other samples, with partial similarity to the current sample. In which case, it would be possible to explore all existing and futuristic samples for entities with a distribution of values, which may, if sampled, improve the current association. To this end, it is possible, for example, to count the number of ambiguities in the current association and look for entities, basic or compound, which have enough values, to resolve these ambiguities in the first place. Of course, it would still be required to check whether co-sampling this new entity in addition to the current entities indeed assists in resolving the ambiguities. Demonstration box 10 presents an illustrative example of this possibility.

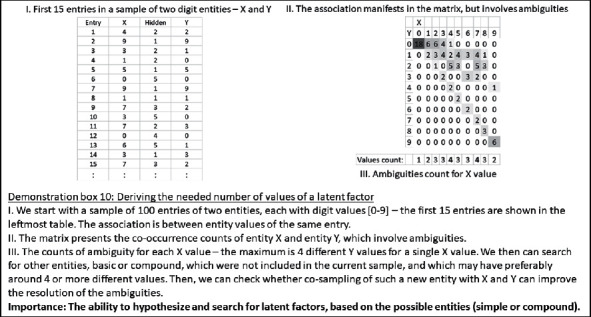

Importantly, we started our analysis from the elementary sensory layer and stated that our entire theory-building process is based on a limited set of basic sensory entities and values. Certainly, as stated, we are capable of generating compound entities. But still, the set of all possible entities and values, which could be candidates as contributors of hidden factors, is limited. In principle, it is possible that some hidden factors would remain beyond our sensory reach and therefore also beyond our theorizing reach.

## 5. Summary

We divided the process of theorizing into three major components: (1) sensation and indexing, (2) bottom-up association, and (3) top-down association. For each major component, we discussed its subcomponents in the brain and with current technology. Thereafter, we described the possible comprehensive expansion of the various subcomponents (except for the first subcomponent of the elementary sensory layer, which could be considered a given, upon which we can build theories). The combination of all these expansions would generate a comprehensive algorithm for associations or theorizing.

As stated, the current manuscript does not claim to suggest an implementational algorithm. It seems that the abstract comprehensive algorithmic components, which are described above, would be, even if feasible to implement, very demanding in terms of computer resources. However, on the one hand, computer resources are constantly improving, and on the other hand, it might be possible to develop practical implementational embodiments of the above described algorithmic components. Furthermore, even a strictly theoretical and impractical description might contribute to our understanding of the theorizing process and the current merits and limitations of its computerized expansions.

We are in an era of ever-growing reliance upon data mining and artificial intelligence for practically any demanding goal, and scientific research is no exception in this regard. Indeed, artificial intelligence and data mining methods produce ever more breakthrough scientific findings. However, with this growing reliance upon such methods, scientists should understand how each such method, or even a combination of methods, is limited in terms of the associations it is able to discover. As was presented in the abstract, the purpose of this manuscript was to offer a computational framework of the theorizing process, as it stems from our brains, as it is currently expanded by technological aids, and as it could be further expanded theoretically to become comprehensive. This may enable the characterization of any state-of-the-art data mining method, in terms of the subcomponents presented here, which it implements, and in terms of its computational limitations even within these sub-components. For example, certain theories might provide effective, yet partial, algorithms, which are mainly limited to specific bottom-up associations [45], while others might provide effective, yet partial, algorithms, which are mainly limited to specific top-down associations [39]. It is beyond the scope of the current manuscript to analyze the various data mining methods extensively. However, if we can produce a framework of the theorizing process, which stems from our neurocognitive abilities in a comprehensive manner, it should be possible to pinpoint in it the scope of the various data mining methods, whether their algorithms are based on cognitive principles, such as symbols manipulation, or on more abstract emergent computation [46].

In all likelihood, the framework that was presented in this manuscript would need significant improvement to meet such a goal. However, in order to offer as sound as possible first step, we tried to set this manuscript upon rather established neurocognitive foundations, which seem to be rather accepted after many decades of brain and behavior research.

Notably, based on the above description, multiple different theories or associations might be identifiable in a given set of samples. Indeed, the preference among these different theories, or association rules, might be defined parametrically in the embodiment of the algorithm. For example, it might be possible to prefer theories, which explain multiple samples, even at the expense of precision and coverage at the single sample level, or alternatively, to prefer precision or degree of single sample coverage (to the level that the top-down component merely serves to fill in some gaps). Altogether, this can promote a more objective quantification of preference among theories according to different parameters. This can also prioritize the use among different possible theoretical constructs [10], which would be the emerging compound entities, identified as useful for the association.
